# Effect of dihydromyricetin on hepatic encephalopathy associated with acute hepatic failure in mice

**DOI:** 10.1080/13880209.2021.1917625

**Published:** 2021-05-13

**Authors:** Long Cheng, Xiaoying Wang, Xueni Ma, Huimei Xu, Yifan Yang, Dekui Zhang

**Affiliations:** Department of Gastroenterology, Lanzhou University Second Hospital, Lanzhou, People’s Republic of China

**Keywords:** Thioacetamide, cognition functions, liver enzymes, glial fibrillary acidic protein, GABAA receptor

## Abstract

**Context:**

Hepatic encephalopathy (HE) is a complex neuropsychiatric disease caused by liver failure. Dihydromyricetin (DMY) is a traditional medicine used to treat liver injury.

**Objective:**

To investigate the effects of dihydromyricetin (DMY) on hepatic encephalopathy associated with acute hepatic failure mice models established by thioacetamide (TAA) exposure.

**Materials and methods:**

Female BALB/c mouse were randomly divided into control, DMY, TAA, and TAA + DMY groups (*n* = 8). The first two groups were intraperitoneally injected with saline or 5 mg/kg DMY, respectively. The last two groups were injected with 600 mg/kg TAA to establish HE models, and then the mice in the last group were treated with 5 mg/kg DMY. Neurological and cognition functions were evaluated 24 and 48 h after injection. Mice were sacrificed after which livers and brains were obtained for immunoblot and histopathological analysis, while blood was collected for the analysis of liver enzymes.

**Results:**

In the TAA + DMY group, ALT and AST decreased to 145.31 ± 12.88 U/L and 309.51 ± 25.92 U/L, respectively, whereas ammonia and TBIL decreased to 415.67 ± 41.91 μmol/L and 3.31 ± 0.35 μmol/L, respectively. Moreover, MDA decreased to 10.74 ± 3.97 nmol/g, while SOD and GST increased to 398.69 ± 231.30 U/g and 41.37 ± 21.84 U/g, respectively. The neurological score decreased to 2.87 ± 0.63, and the number of GFAP-positive cells decreased to 41.10 ± 1.66. Furthermore, the protein levels of TNF-α, IL-6, and GABA_A_ in the cortex decreased.

**Conclusions:**

We speculate that DMY can serve as a novel treatment for HE.

## Introduction

Acute liver failure is a severe form of liver damage caused by various factors including viral and autoimmune hepatitis, hepatic ischaemia, paracetamol toxicity, and drug-induced liver injury caused by herbal and prescription drugs (Stravitz and Lee 2019). It is an infrequent but life-threatening disease because many factors that cause damage to hepatocytes results in elevation of aminotransferases, disturbed coagulation, and altered mentation (Squires et al. 2018). Hepatic encephalopathy (HE) is one of the most serious complications of acute hepatic failure, and severe hepatic encephalopathy in patients with cirrhosis is associated with a mortality rate of more than 50% in the first year alone. HE manifests as a spectrum of neuropsychiatric abnormalities. Recent studies have shown that neuroinflammation, neurotransmitter function, oxidative stress, hyperammonemia, and the blood-brain barrier modulate the pathogenesis of hepatic encephalopathy (Wijdicks 2016). It is worth noting that most HE drugs aim at reducing ammonia levels. However, the drugs are limited by a lack of treatment for other HE precipitating factors.

Accumulating evidence has confirmed that dihydromyricetin (DMY), the most abundant natural flavonoid in rattan tea, has a wide spectrum of pharmacological effects. Beyond the general characteristics of flavonoids, DMY has hepatoprotection, neuroprotection, dermatoprotection, cardioprotection, antidiabetes, and antitumor effects (Liu et al. 2017). A recent study has reported that supplementation with DMY effectively ameliorated the development of NAFLD by inhibiting the accumulation of liver lipids. The researchers attributed these hepatoprotective effects to DMY’s antioxidant capacity (Zhang et al. 2018). Therefore, we hypothesised that DMY can alleviate hepatic encephalopathy through hepatoprotection. This study will investigate the functions of DMY in HE model mice established by hepatotoxin thioacetamide (TAA) exposure and elucidate the underlying mechanisms.

## Materials and methods

### Mice

Six- to eight-week-old female BALB/c mice (18–22 g, provided by the Experimental Animal Centre of Lanzhou University) were randomly assigned to four different groups (*n* = 8). The mice were acclimatised and housed in cages containing woodchip bedding under standard temperature and humidity conditions (temperature: about 22 °C; relative humidity: about 50%), in a pathogen-free room on a 12 h light/dark cycle (lights on at 8 a.m.) with free access to food and water. All procedures involving animals and their care strictly complied with the Animal Research Institute Committee guidelines of the Lanzhou University Second Hospital (D2019-084). Moreover, all procedures were performed under anaesthesia to minimise suffering.

### Hepatic failure model

Mice hepatic failure models were established by intraperitoneal injection of 600 mg/kg thioacetamide (TAA, Sigma-Aldrich, USA), which was dissolved in sterile normal saline (NS) solution. Twenty-four hours after TAA injection, all animals were injected (subcutaneously) with 0.5 mL solution of 0.45% NaCl, 0.2% KCl, and 5% dextrose in order to prevent electrolyte disturbances and hypoglycaemia (Avraham et al. 2011).

### DMY administration

DMY (Chengdu Must Biotechnology Co., Ltd, CN) was dissolved in heated saline, followed by intraperitoneal injection of a single dose of 5 mg/kg one day after TAA treatment.

### Neurofunctional assessment

Neurological function was determined using a 10-point scale based on task performance and reflexes: climbing onto a round and a square pole, balancing on a beam 3, 2, and 1 cm in width, exit from a circle in less than 3 min, hemiparesis, startle reflex, walking on a straight line, and seeking behaviour (Stahel et al. 2000). A score of 1 was assigned for each abnormal reflex reaction or failed task. Therefore, the higher the score, the poorer the neurological function. We evaluated the neurological score one day after TAA treatment, and then the animals were divided into two groups: untreated and treatment groups, which were injected with normal saline (NS) or DMY, respectively. Next, we evaluated the neurological score of the two groups one day after injection.

### Cognitive function

Spatial learning and memory function were assessed using the Morris water maze test (Zhan et al. 2019). Briefly, mice were trained four times each day for five consecutive days in a circular pool where each mouse was permitted 60 s to find the hidden platform, which was 10 cm in diameter and submerged 1 cm below the water surface in the target quadrant. If the mice failed to locate the platform, they were guided to the platform and remained there for 15 s in order to ensure that each mouse could successfully find the platform after training. On the sixth day, we removed the platform and subjected the mice to a 60 s probe trial, which was recorded and used to evaluate reference spatial memory. We used a digital video camera (WMT-100S Morris, Chengdu Taimeng Software Co., Ltd, CN) to record the number of times the mice crossed the platform area (platform crossing).

### Liver histopathology

The mice were sacrificed by decapitation two days after treatment with TAA, followed by immediate removal of the livers for haematoxylin and eosin (H&E) staining. Firstly, the liver tissue was harvested and fixed with 10% formalin solution. Approximately 24 h later, the liver was embedded in liquid paraffin and sectioned into 6 µm sections for H&E staining. Finally, we observed the pathological changes under a light microscope, and randomly selected 20–30 views from each side at a magnification of 200×.

### Biochemical analyses

Serum for alanine transaminase (ALT), aspartate transaminase (AST), albumin, bilirubin, and ammonia were quantified using an automated clinical chemistry analyser (Cobas, Roche).

### Hepatic biochemical parameters

The MDA and GST levels and the activity of SOD were measured by absorbance. All assay procedures were carried out according to the instruction manual (Solarbio, CN).

### Immunohistochemistry of the hippocampus

Mice were sacrificed on the 3rd day (48 h after TAA injection) after model establishment. We removed the brain and cut it into two pieces along the midline, followed by fixation with 10% paraformaldehyde. After 24 h, the brain was embedded in liquid paraffin and sectioned into 6 µm sections. Next, the sections were used for immunohistochemical staining of glial fibrillary acidic protein (GFAP) according to a standard protocol. Paraffin-embedded brain sections were deparaffinized, rehydrated, and boiled three times in 10 mM citrate buffer at pH 6. All sections were then incubated with hydrogen peroxide (0.3% in phosphate buffer saline) in order to quench endogenous peroxidase, followed by incubation with blocking buffer (goat serum) for 1 h. Some sections were selected and incubated with the primary antibody against GFAP (1:2500, DakoCytomation, DK) overnight at 4 °C. The tissue sections were then washed three times with PBS and incubated with secondary antibody (goat anti-rabbit, 1:300, Vectorlabs, USA) for 30 min. Later, the sections were washed three times in PBS and the avidin-biotin immunoperoxidase reaction visualised using diaminobenzidine (DAB) (Vectorlabs, USA). Finally, the sections were counterstained with haematoxylin and observed under a light microscope (Zeiss, German). Images were then captured using a digital camera (OLYMPUS, Japan) mounted directly on the microscope. The activation of astrocytes was assessed in each hippocampal section of both hemispheres, where cells were assessed in 5–7 random visual fields per hemisphere. We counted 100 cells in each field, and calculated the percentage of GFAP-positive astrocytes and staining intensity according to a previously described protocol (Fromowitz et al. 1987).

### Western blot analysis

Protein levels of GABA_A_, TNF-α, and IL-6 in the cerebral cortex were determined using immunoblot analysis. Firstly, the tissues were homogenised in RIPA lysis buffer and centrifuged at 13,000 rpm for 30 min at 4 °C. The total protein concentration in the supernatant was determined using BCA protein assay kits (Thermo Scientific, USA). The proteins were resolved on 10% sodium dodecyl sulfate-polyacrylamide gels and transferred onto polyvinylidene difluoride membranes. The membrane was blocked with blocking solution for 1 h at room temperature and then incubated with the appropriate primary antibody overnight at 4 °C (anti-GABARα, 1:1000, Proteintech, USA; anti-IL-6, 1:1000, Proteintech, USA; anti-TNF-α, 1:1000, Proteintech, USA; and anti-GAPDH, 1:10000, Proteintech, USA). After three washes in TBST, the blots were incubated with the secondary antibodies (1:2500, Proteintech, USA) for 1 h at room temperature. A fluorescence scanner (Automatic Gel Imaging Analysis System, Peiqing Science and Technology CO., Ltd, CN) was then used to visualise the bands.

### Experimental design

#### Experiment 1

The mice were divided into four groups (*n* = 8), A (Control), B (DMY), C (TAA), and D (TAA+*DMY*). Mice in group C and D were administered with TAA (600 mg/kg) on the first day of the experiment, while group B mice were treated with DMY (5 mg/kg) and group A with saline as the control. We assessed the neurological function 24 h after injection, and then administered group D mice with DMY (5 mg/kg). The other groups were not treated. All animals were injected (subcutaneously) with 0.5 mL of a solution containing 5% dextrose, 0.45% NaCl, and 0.2% KCl to prevent electrolyte disturbances. On the third day, we assessed the neurological performance again, and sacrificed all the mice. Blood was drawn for serum analysis, while livers and brains were fixed in 10% formaldehyde for histopathological observation.

#### Experiment 2

The mice were grouped as described in Experiment 1. They were then subjected to the Morris water maze test where they were trained four times each day for five consecutive days in a circular pool. During training, each mouse was permitted 60 s to find the platform. On the sixth day, a 60 s probe trial was implemented and the number of times a mouse crossed the platform area were recorded. Finally, all mice were sacrificed by cervical dislocation, and their livers and brains were removed for liver enzyme testing and immunoblot of the brain, respectively.

## Statistical analysis

SPSS software was used for statistical analyses, and all data were expressed as mean ± SD (standard deviations). Kruskal–Wallis *H* test was used to analyse the cognitive function. All other experimental results were analysed using one-way analysis of variance (ANOVA), followed by the Newman–Keuls Multiple Comparison Test.

## Results

### Neurological score

**Figure 1.**
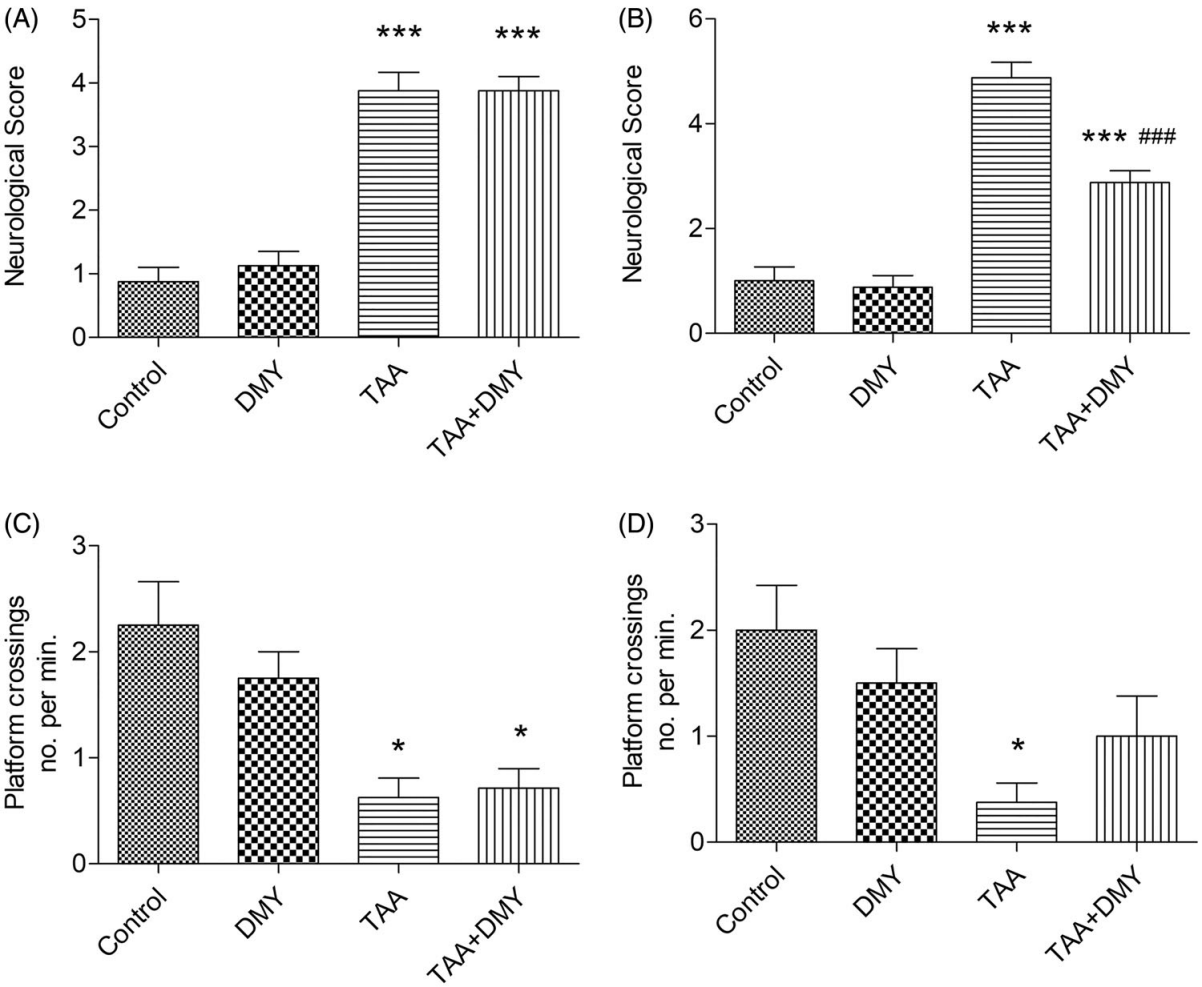
Neurological and cognitive function of HE mice. (A) Neurological score of mice 24 h after TAA injection. (B) Neurological score of mice 24 h after DMY injection. (C) Cognitive function of mice 24 h after TAA injection. (D) Cognitive function of mice 24 h after DMY injection. DMY: DMY-treated (5 mg/kg) group; TAA: TAA-treated (600 mg/kg) group; TAA + DMY: TAA-treated (600 mg/kg) + DMY-treated (5 mg/kg) group. Values are expressed as mean ± SD (*n* = 8). *p < 0.05 and ***p < 0.001 vs. control; ###*p* < 0.001 vs. TAA.

TAA significantly increased the neurological score of mice compared to the control group (Figure 1(A), 3.88 ± 0.84 vs. 0.87 ± 0.64, *p* < 0.001). In addition, the neurological score was improved in TAA-treated mice after administration of DMY (5 mg/kg) compared to TAA treatment alone (Figure 1(B), 2.87 ± 0.63 vs. 4.91 ± 0.82, *p* < 0.001). DMY treatment did not affect the score of control animals.

### Cognitive function

Results indicated that the cognitive function was significantly impaired following TAA exposure, where HE mice exhibited a significant decrease in the number of times they crossed the platform area (Figure 1(C)). However, the impaired cognitive function was partially restored after DMY treatment (Figure 1(D)).

### Liver histopathology

**Figure 2.**
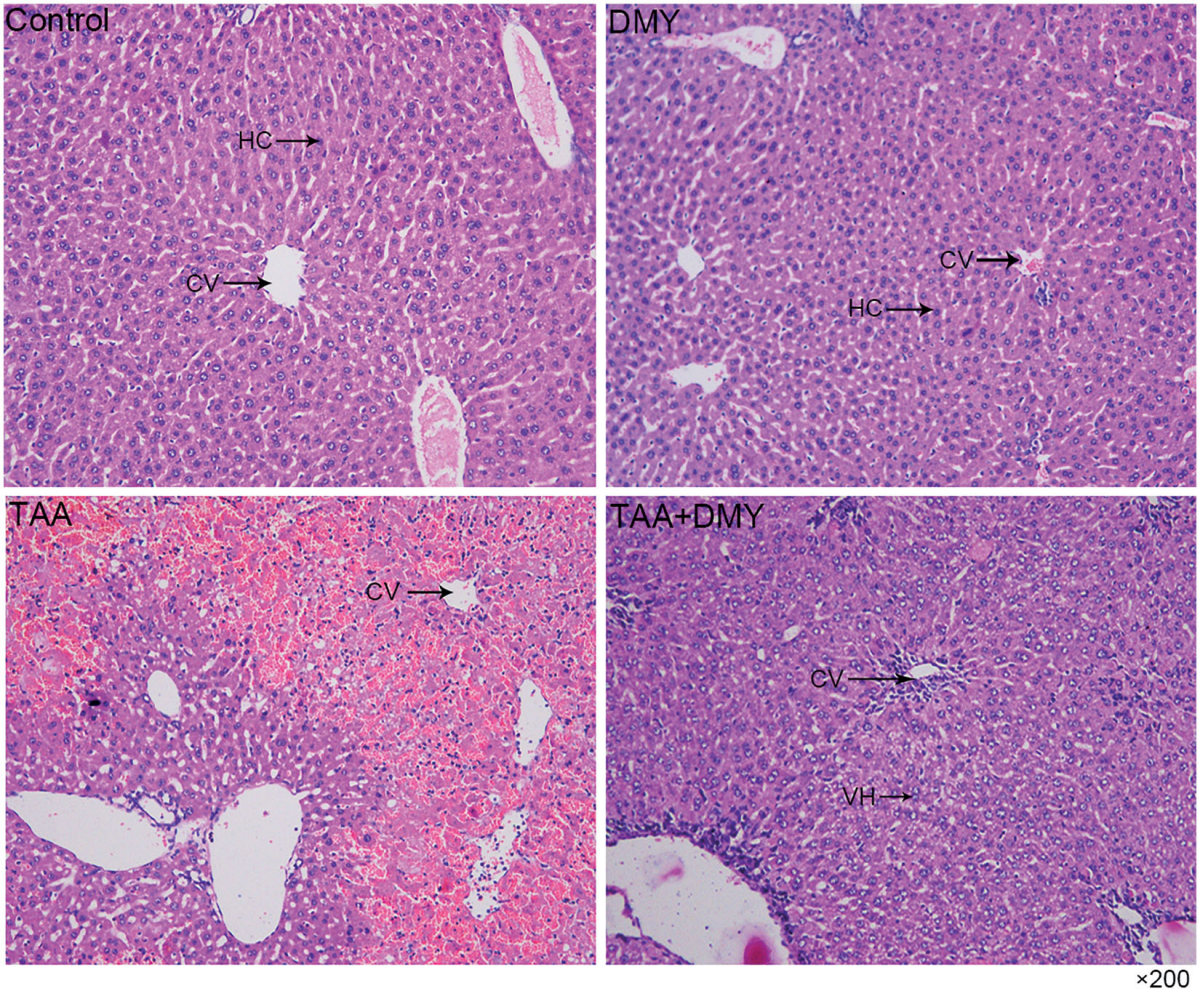
H&E staining of the liver (200×). The histopathology of control and DMY groups shows normal liver architecture. In TAA group, the liver shows a massive hepatocellular necrosis in some part of the liver; the necrosis is focal but not diffuse. In the TAA + DMY group, hepatocytes were hypertrophied with many cytoplasmic vacuoles and oedematous enlarged nuclei, indicating hepatocellular degeneration. Mild mononuclear infiltration was also observed around the central vein. Hepatocellular injury is clearly milder in the TAA + DMY than the TAA group. HC: hepatocytes; CV: central vein; VH: vacuolated hepatocytes.

Histopathological examination of control and DMY groups showed normal architecture of the liver. No vacuoles, swelling, or necrosis of liver cells was observed. Group C mice showed a massive hepatocellular necrosis in some part of the liver after TAA treatment; the necrosis is focal but not diffuse. In group D (TAA + DMY), hepatocytes were hypertrophied with many cytoplasmic vacuoles and oedematous enlarged nuclei, indicating hepatocellular degeneration. Moreover, mild mononuclear infiltrate was observed around the central vein (Figure 2).

### Liver biochemistry

**Figure 3.**
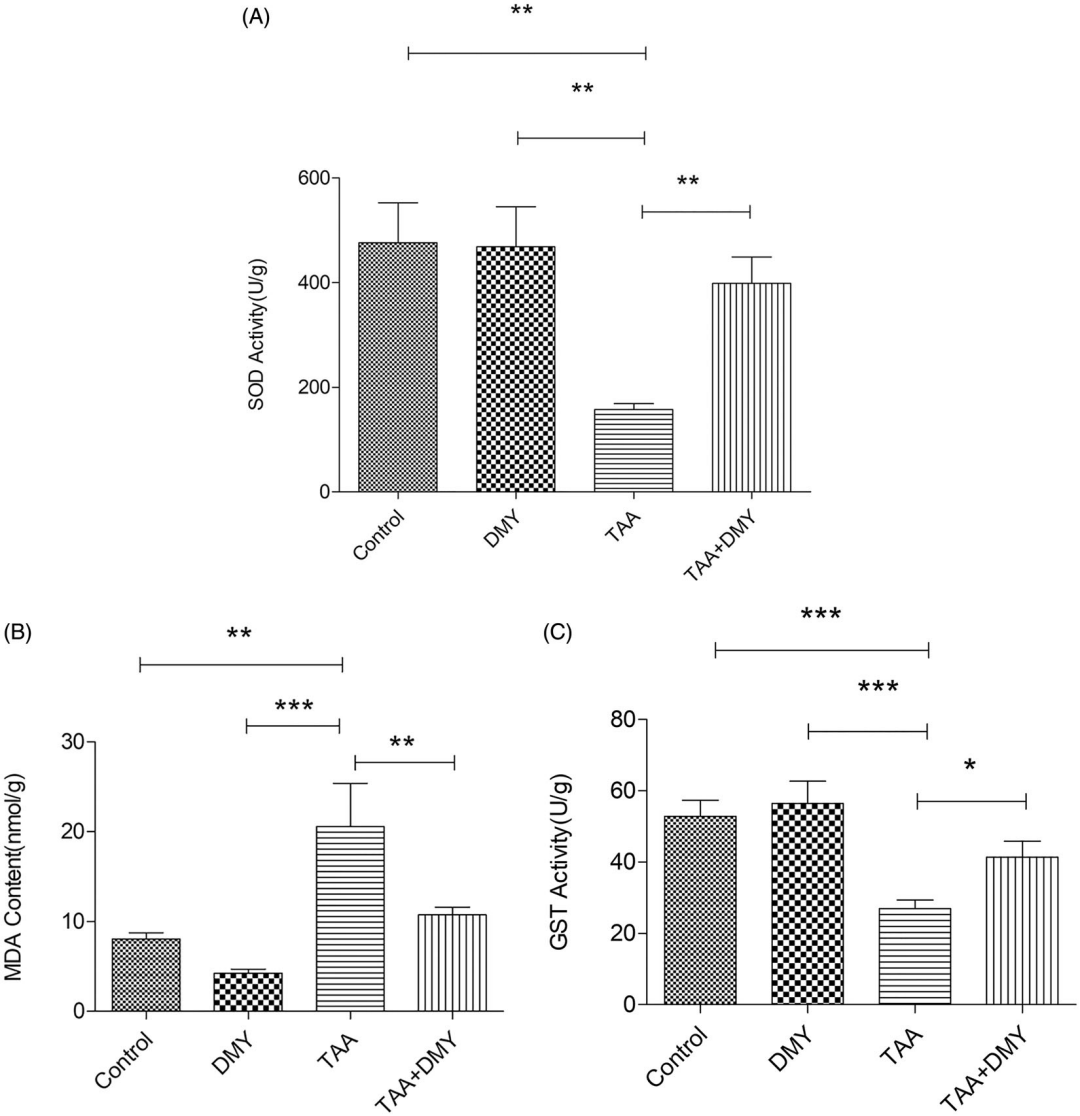
Liver function. (A) The level of SOD. (B) The level of MDA. (C) The level of GST. DMY: DMY-treated (5 mg/kg) group; TAA: TAA-treated (600 mg/kg) group; TAA + DMY: TAA-treated (600 mg/kg) + DMY-treated (5 mg/kg) group. Values are expressed as mean ± SD (*n* = 8). *p < 0.05, **p < 0.01, and ***p < 0.001.

The levels of ammonia, bilirubin, albumin, and liver enzymes (AST and ALT) were increased after TAA treatment. On the other hand, DMY administration significantly decreased the level of bilirubin, AST, and ALT compared to the untreated mice (Table 1). Moreover, the level of MDA was also increased after TAA administration and DMY administration significantly decreased its level (Figure 3(B), 20.55 ± 23.70 vs. 10.74 ± 3.97, *p* < 0.01). On the contrary, the levels of SOD and GST were decreased after TAA administration, but the levels were raised after DMY treatment (Figure 3(A), 157.13 ± 53.45 vs. 398.69 ± 231.30, *p* < 0.01; Figure 3(C) 26.94 ± 11.21 vs. 41.37 ± 21.84, *p* < 0.05). It is worth noting that DMY treatment had no effect on the levels of these indicators in control animals.

**Table 1.** Hepatic biochemistry.

Group	Control	DMY	TAA	TAA + DMY
Ammonia (umol/L)	191.90 ± 30.82	191.43 ± 30.37	437.13 ± 43.42*	415.67 ± 41.91*#
ALT (U/L)	66.43 ± 18.67	67.53 ± 18.74	224.80 ± 27.83*	145.31 ± 12.88*⋆
AST (U/L)	110.82 ± 30.68	101.04 ± 27.62	488.47 ± 47.92*	309.51 ± 25.92*⋆
TBIL (umol/L)	1.77 ± 0.36	1.74 ± 0.46	5.23 ± 0.65*	3.31 ± 0.35*⋆
ALB (g/L)	30.10 ± 2.2	29.87 ± 2.43	27.69 ± 5.57※	30.06 ± 3.35※#

DMY: DMY-treated (5 mg/kg) group; TAA: TAA-treated (600 mg/kg) group; TAA + DMY: TAA-treated (600 mg/kg)+DMY-treated (5 mg/kg) group. Values are expressed as mean ± SD (*n* = 8). ※*p* > 0.05 and *p < 0.001 vs. control; #*p* > 0.05 and ⋆*p* < 0.001 vs. TAA.

### Immunohistochemistry of the hippocampus

**Figure 4.**
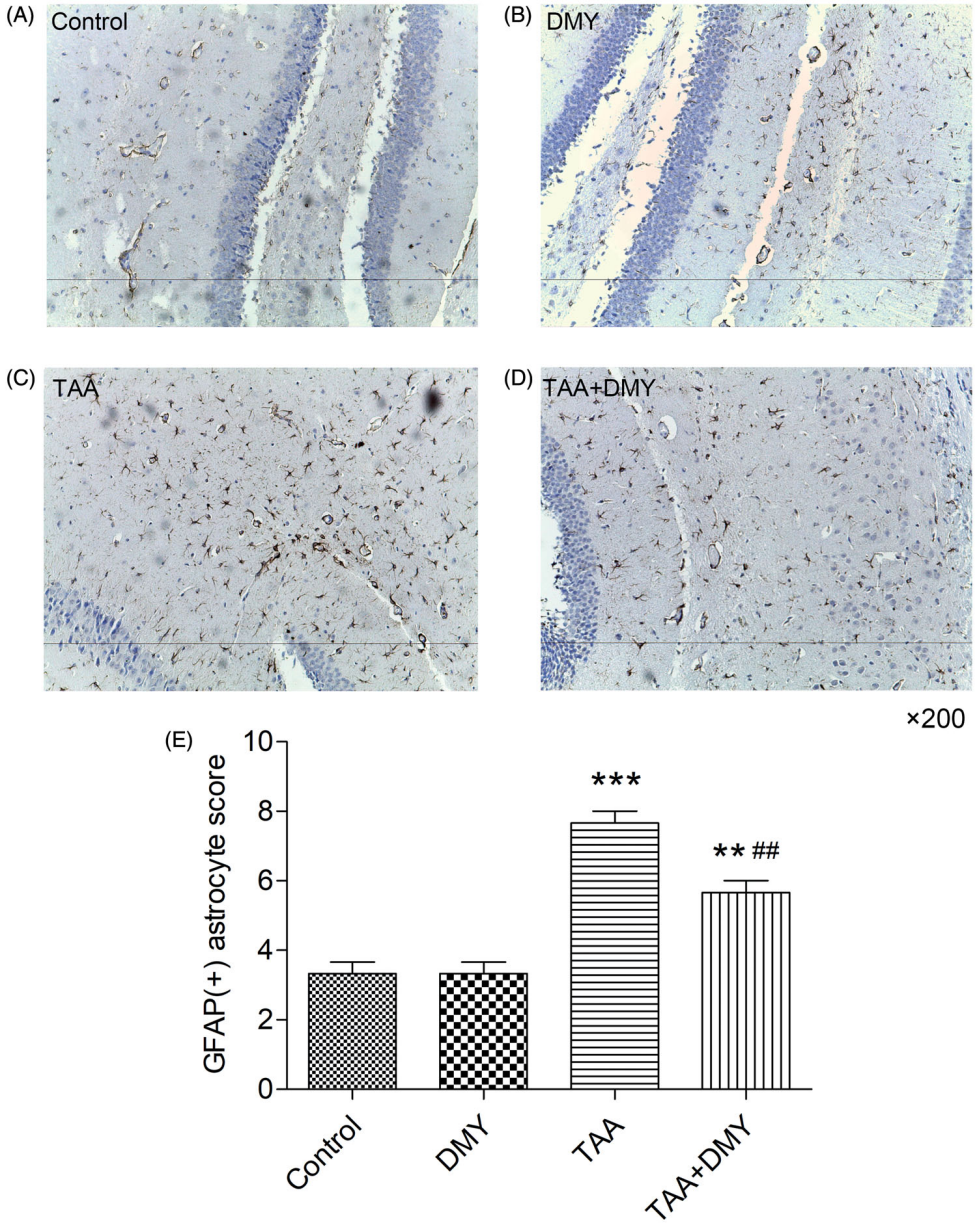
Immunohistochemistry of the hippocampus (200×). GFAP (+) astrocytes were not obvious in naive controls (A, B), but they were significantly increased in TAA-treated mice (C, D). DMY administration had no effect on the activation of astrocytes in group B. DMY treatment significantly reduced the number of activated astrocytes in group D compared to group C. (E) Hippocampus GFAP (+) astrocyte score was quantified. DMY: DMY-treated (5 mg/kg) group; TAA: TAA-treated (600 mg/kg) group; TAA + DMY: TAA-treated (600 mg/kg) + DMY-treated (5 mg/kg) group. Values are expressed as mean ± SD (*n* = 8). **p < 0.01 and ***p < 0.001 vs. control; ##*p* < 0.01 vs. TAA.

Figure 4 shows the immunohistochemistry of brain tissue. Astrocyte activation was observed in TAA-treated mice and it was apparent that the number of GFAP-positive cells increased significantly compared to the control (Figure 4(A,C), 6.30 ± 3.43 vs. 48.20 ± 2.62, *p* < 0.01). The parameter was unaffected in DMY-treated controls. In addition, the number of GFAP-positive cells was decreased after administration of DMY (5 mg/kg) compared to TAA treatment alone (Figure 4(C,D), 41.10 ± 1.66 vs. 48.20 ± 2.62, *p* < 0.01). Hippocampus GFAP (+) astrocyte score was increased in TAA-treated mice compared to the control group but it decreased after DMY administration (Figure 4(E)). These results suggest that TAA treatment increased the number of activated astrocytes, but this effect was significantly reduced after DMY administration.

### Western blot analysis

**Figure 5.**
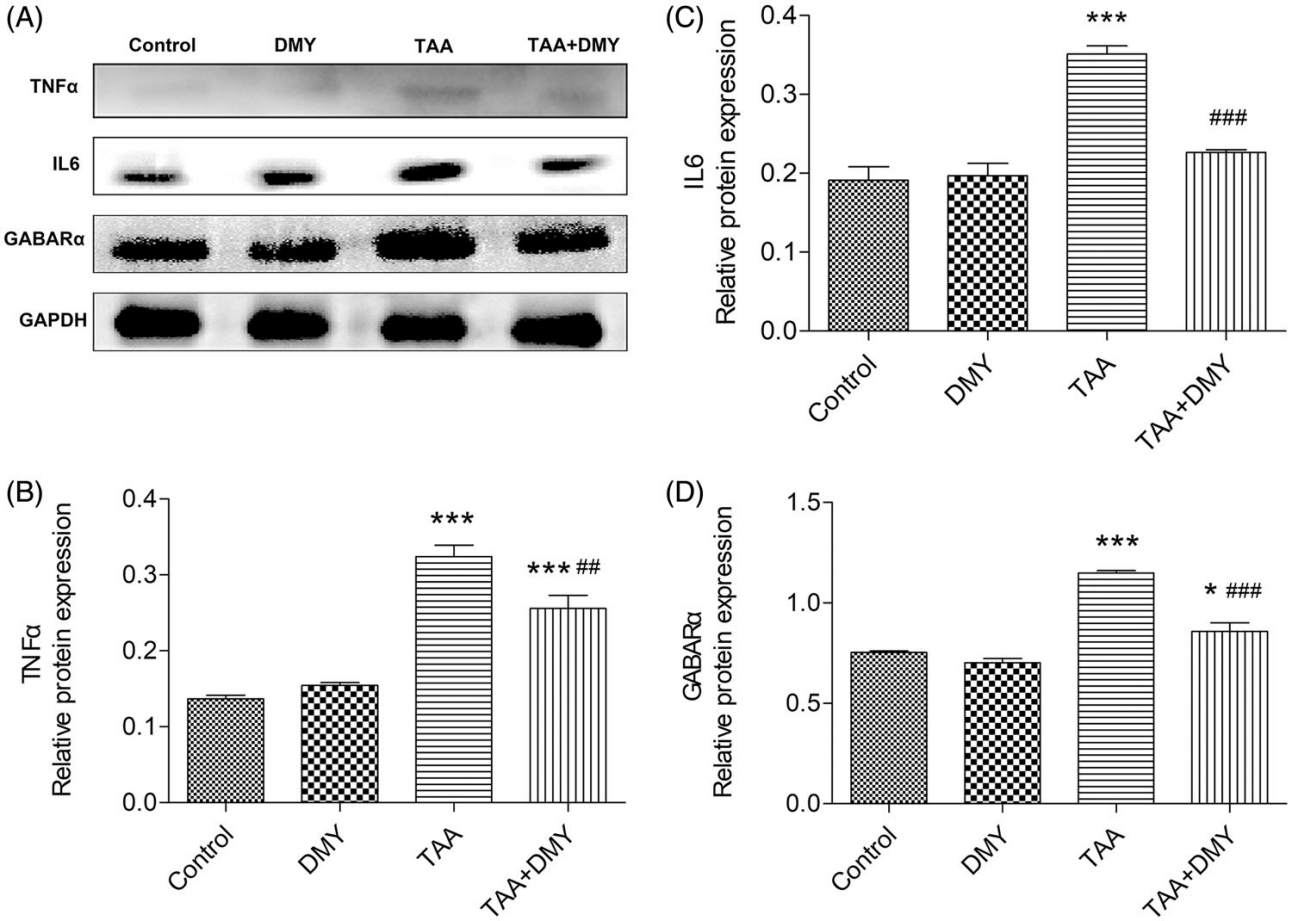
Western blot analysis. (A) Protein levels of TNF-α, IL-6, and GABA_A_ in the cortex. Relative protein expression was quantified using Image J. (B) Relative protein expression of TNF-α. (C) Relative protein expression of IL-6. (D) Relative protein expression of GABA_A._ DMY: DMY-treated (5 mg/kg) group; TAA: TAA-treated (600 mg/kg) group; TAA + DMY: TAA-treated (600 mg/kg) + DMY-treated (5 mg/kg) group. Values are expressed as mean ± SD (*n* = 8). *p < 0.05 and ***p < 0.001 vs. control; ##*p* < 0.01 and ###*p* < 0.001 vs. TAA.

Western blots revealed that TAA treatment increased the protein levels of TNF-α, IL-6, and GABA_A_ in the cortex compared to the control. However, the parameters were significantly decreased after DMY administration in TAA-treated mice (Figure 5).

## Discussion

HE is caused by a combination of different pathophysiological mechanisms such as impaired blood-brain barrier (BBB) permeability, neurotoxins, oxidative stress, inflammation, and impaired energy metabolism by the brain (Hadjihambi et al. 2018). Generally, HE treatment depends on its severity, and most drugs target ammonia reduction and suppressing neurotoxin production in the bowel. However, the treatment is limited by the fact that it does not address other precipitating factors involved in HE development such as inflammation, oxidative stress, or other cerebral alterations. DMY possesses diverse biological and pharmacological activities and is conventionally used as an antioxidant and anti-inflammatory agent (Liu et al. 2019). A previous study reported that DMY can suppress oxidative stress in the hippocampus of T2DM mice by increasing the activities of all antioxidants including SOD, CAT, and GSH-PX (Ling et al. 2018). In another study, MDA, a metabolite of lipid peroxidation, was the main index for reactive oxygen species which was decreased after DMY treatment (Song et al. 2017). Besides, DMY can also attenuate the mRNA and protein levels of TNF-α and IL-6 in LPS-induced neuroinflammation in the hippocampus (Ren et al. 2018). A recent study has shown that DMY treatment improved steatosis, inflammation, and fibrosis, which are three main aspects of non-alcoholic steatohepatitis and some of the metabolic basal characteristics (Zeng et al. 2020). Similar results were obtained in this study where DMY had a neuroprotective role in TAA-treated mice. We found that it restored liver function, normalised the levels of inflammatory factors, and improved the liver and brain pathology. Furthermore, another study suggested that DMY exerts an anti-inflammatory effect by suppressing the activation of NF-κB and the phosphorylation of JNK and p38 (Hou et al. 2015). Given that there are many inflammatory signalling pathways, researchers should determine whether DMY can also play a role through other pathways.

It is speculated that astrocyte swelling is closely associated with the accumulation of intracellular glutamine because astrocytes take up ammonia and convert it to glutamine. Ultimately, astrocyte swelling results in neuronal dysfunction. Moreover, the swelling induces oxidative stress and forms reactive oxygen species, thereby further aggravating astrocyte swelling. The glutamine produced by astrocytes forms an osmotic gradient since it is an osmotically active molecule, eventually causing cerebral edoema (Parekh and Balart 2015). A recent study reported that ammonia toxicity and HE are associated with premature astrocyte senescence, which may impair neurotransmission and contribute to the persistence of cognitive disturbances (Gorg et al. 2015). In addition, ammonia and inflammatory agents can activate the toll-like receptor 4 (TLR4) in endothelial cells (ECs), ultimately causing astrocyte swelling (Jayakumar et al. 2014). Besides, the mechanisms underlying astrocyte swelling may also be associated with glial fibrillary acidic protein, glutamate, alanine, lactate, glutathione, manganese, epidermal growth factor receptor, 18 KDa translocator protein, and aquaporin-4 water channel, as well as oxidative stress, inflammation, mitochondrial permeability transition, and ATP depletion (Sepehrinezhad et al. 2020). GFAP, the principal intermediate filament (IF) protein of astrocytes, is involved in physiological and pathophysiological functions of astrocytes (Hol and Pekny 2015). In this study, we have shown that astrocytes were significantly activated in HE mice, indicating the importance of astrocytes in brain edoema.

Several recent studies have shown that inflammation including systemic inflammation, neuroinflammation, and endotoxemia, modulates HE pathogenesis (Luo et al. 2015). A previous study reported that portacaval shunt (PCS) rats exhibited increased activities of cyclooxygenase and inducible NO synthase, and increased levels of IL6 in the cerebral cortex, indicating the presence of neuroinflammation. Subsequent treatment with ibuprofen, an anti-inflammatory drug, normalised the activities of cyclooxygenase and inducible NO synthase, and completely restored the cognitive ability of the rats (Cauli et al. 2007). Another study demonstrated the importance of neuroinflammation and revealed that neuroinflammation could be caused by peripheral inflammation. It was observed that an increase in the expression of TNF-α, IL-1b, and nuclear NF-κB in the hippocampus of HE rats established by PCS exposure led to altered neurotransmission and finally impaired spatial learning and memory. However, administration of anti-TNF-α drugs, which could not cross the blood-brain barrier, reduced neuroinflammation, translocated NF-κB to the nucleoli, and normalised TNF-α and IL-1b in the hippocampus. Ultimately, spatial learning and memory were restored (Dadsetan et al. 2016). Similar results were obtained in this study where the expression of TNF-α and IL-6 were increased in the brain, suggesting that inflammatory factors modulate brain edoema caused by hepatic encephalopathy. Moreover, DMY normalised the expression of TNF-α and IL-6.

Hepatic encephalopathy is a severe neuropsychiatric complication in patients suffering from acute or chronic liver failure. Furthermore, the pathogenesis of HE has not yet been fully elucidated. However, the increase of γ-aminobutyric acid (GABA) level and GABAergic activity is considered to be one of the important factors since GABA is one of the most important inhibitory neurotransmitters in the central nervous system (CNS). A previous study reported that GABA synthesis was enhanced in BDL rat model of chronic HE compared to sham-operated animals (Leke et al. 2011). The structures and functions of three types of GABA receptors have been discovered, namely GABA_A_, GABA_B,_ and GABA_C_. Among them, accumulating evidence indicates a close relationship between the GABA_A_ receptor and HE. One study reported that the expression of GABA_A_ receptor subunits (α1 and β1) was significantly increased in hippocampi, basal nuclei, substantianigra pars reticularis, and substantia nigra pars compacta of HE model rats (Li et al. 2005). Another study also showed that the membrane expression of GABA receptors was strongly altered in hyperammonemic rats, and the α-1 subunit of GABA receptors was selectively increased (Hernandez-Rabaza et al. 2016). Similarly, our Western blot results indicated that α subunits of GABA_A_ receptors were up-regulated in TAA-treated mice. However, DMY treatment decreased the expression. Furthermore, the β subunit of GABA_A_ receptors was also considered to be involved in hepatic encephalopathy (Ding et al. 2018). This suggests that both subunits function in the pathogenesis of hepatic encephalopathy.

## Conclusions

This study revealed that DMY possesses a therapeutic effect on TAA-induced acute HE model because it restored both liver function and brain histopathology, thereby leading to the improvement of HE symptoms. Therefore, we speculate that DMY could serve as a novel therapeutic against HE, our work also sheds new light on complementary and alternative therapy for HE.
